# Editorial: Chronic pain: what is the mechanism?

**DOI:** 10.3389/fphar.2024.1496510

**Published:** 2024-10-31

**Authors:** James David Adams, Ching-Liang Hsieh, Roberta Gualdani

**Affiliations:** ^1^ Independent researcher, Benicia, CA, United States; ^2^ Hsieh Acupuncture Research Center, China Medical University, Taichung, Taiwan; ^3^ Gualdani Fonds National de la Recherche Scienfitique, Université Catholique de Louvain, Louvain-la-Neuve, Belgium

**Keywords:** pain, chronic pain, chemokine, transient receptor potential cation channel, prostaglandin

Chronic pain reportedly affected 20.9% of adults in the US in 2021 or 51.6 million people, with 17.1 million of them suffering severe chronic pain that altered the ability to function normally (Ricard et al., 2023). Chronic pain is routinely treated with oral nonsteroidal anti-inflammatory agents that include acetaminophen, aspirin, ibuprofen, and naproxen (Dydyk et al., 2024). These agents kill about 50,000 thousand patients yearly from ulcers, strokes, and heart attacks (Adams, 2020; Adams, 2017). Opioids are the drugs of second choice (Dydyk et al., 2024) and kill at least 75,000 people in the US yearly from respiratory depression and seizures, as reported by the Centers for Disease Control on 17 November 2021. Other drugs can be used, such as antiepileptics, antidepressants, and muscle relaxers (Dydyk et al., 2024). None of these drugs cures chronic pain. This is because the mechanism of chronic pain is not clearly understood. A clear understanding of the mechanism may lead to more effective therapy.

Long et al., in this Research Topic, have published their work on bufalin-PLGA as a form of treatment for neuropathic pain. Bufalin is derived from toad venom and was found to decrease the levels of IL-1β, IL-18, and TNFα during chronic pain. The expression of transient receptor potential cation channels of the vanilloid 1 type (TRPV1) and purinergic 2X7 receptors increased during chronic pain and decreased after bufalin administration. These receptors are important in pain and chronic pain. IL-1β and IL-18 increase the synthesis and release of several chemokines (Burke et al., 2015; Fehniger et al., 1999). TNFα also stimulates the synthesis and release of chemokines (Ganesan et al., 2002). By decreasing chemokine production, bufalin may alleviate chronic pain. Chemokines have been implicated in a pain chemokine cycle that may be involved in chronic pain (Adams, 2020; Adams, 2017).

Shen et al. reviewed the mechanisms of inflammation in retinal diseases. IL 1β, IL-6, IL-10, and IL-12 levels increase in these conditions and may be involved in causing hyperpermeability of the choroid, leading to edema and inflammation. These cytokines increase the release of chemokines, including MCP-1 and IL-8, which attract inflammatory cells such as macrophages, T cells, and B cells into the retina (Burke et al., 2015; Fehniger et al., 1999). Dendritic cells also become activated due to the actions of these chemokines. Immunotherapy development is ongoing to find ways to decrease the actions of these inflammatory chemokines.

Kuo et al. investigated a new somatostatin type 4 receptor agonist that alleviates pain, chronic pain, and inflammation. Somatostatin agonists are known to act on inflammatory macrophages and decrease the secretion of IL-8 and MCP-1 by these macrophages (Armani et al., 2006). These chemokines are important in pain and inflammation (Adams, 2020; Adams, 2017). This may be involved in the alleviation of pain, chronic pain, and inflammation by J-2156.

Heinle et al. discussed the importance of NaV1.8 channels in chronic pain. NaV1.8 is expressed in several tissues including the brain, brain stem, and skin (Zhang et al., 2022). The receptor is important in pain sensation and complements TRP channels, which are the most abundant pain receptors. Chemokines, such as CCL2, are known to increase NaV1.8 expression in chronic pain (Belkouch et al., 2011), similar to chemokines increasing TRP channel expression in chronic pain (Adams, 2020; Adams, 2017). It is important to remember that visceral neurons have peripheral projections into the skin, which is where much of the visceral pain is sensed in NaV1.8 receptors and TRP channels (Adams, 2020; Adams, 2017).

In this Research Topic, the importance of the pain chemokine cycle is elucidated. Chemokines increase pain by activating and inducing pain receptors and attracting inflammatory cells. Macrophages are attracted by chemokines and produce prostaglandins since they express cyclo-oxygenase 2. Prostaglandins interact with prostaglandin receptors to cause pain and transactivate TRP channels to cause more pain and chemokine release (Adams, 2020; Adams, 2017). Neutrophils are attracted by chemokines to sites of pain and inflammation and secrete leukotrienes that cause long-term activation of TRP channels and pain (Adams, 2020; Adams, 2017). This pain chemokine cycle is largely located in the skin and can last for many years.
